# Sphingolipids in Human Synovial Fluid - A Lipidomic Study

**DOI:** 10.1371/journal.pone.0091769

**Published:** 2014-03-19

**Authors:** Marta Krystyna Kosinska, Gerhard Liebisch, Guenter Lochnit, Jochen Wilhelm, Heiko Klein, Ulrich Kaesser, Gabriele Lasczkowski, Markus Rickert, Gerd Schmitz, Juergen Steinmeyer

**Affiliations:** 1 Department of Orthopedics, Justus-Liebig-University Giessen, Giessen, Germany; 2 Department of Clinical Chemistry and Laboratory Medicine, University Hospital Regensburg, Regensburg, Germany; 3 Department of Biochemistry, Justus-Liebig-University Giessen, Giessen, Germany; 4 Medical Clinic II/IV, Justus-Liebig-University Giessen, Giessen, Germany; 5 Internistisches Praxiszentrum am Krankenhaus Balserische Stiftung, Giessen, Germany; 6 Institute of Forensic Medicine, Justus-Liebig-University Giessen, Giessen, Germany; University of Leuven, Rega Institute, Belgium

## Abstract

Articular synovial fluid (SF) is a complex mixture of components that regulate nutrition, communication, shock absorption, and lubrication. Alterations in its composition can be pathogenic. This lipidomic investigation aims to quantify the composition of sphingolipids (sphingomyelins, ceramides, and hexosyl- and dihexosylceramides) and minor glycerophospholipid species, including (lyso)phosphatidic acid, (lyso)phosphatidylglycerol, and bis(monoacylglycero)phosphate species, in the SF of knee joints from unaffected controls and from patients with early (eOA) and late (lOA) stages of osteoarthritis (OA), and rheumatoid arthritis (RA).

SF without cells and cellular debris from 9 postmortem donors (control), 18 RA, 17 eOA, and 13 lOA patients were extracted to measure lipid species using electrospray ionization tandem mass spectrometry - directly or coupled with hydrophilic interaction liquid chromatography.

We provide a novel, detailed overview of sphingolipid and minor glycerophospholipid species in human SF. A total of 41, 48, and 50 lipid species were significantly increased in eOA, lOA, and RA SF, respectively when compared with normal SF.

The level of 21 lipid species differed in eOA SF versus SF from lOA, an observation that can be used to develop biomarkers. Sphingolipids can alter synovial inflammation and the repair responses of damaged joints. Thus, our lipidomic study provides the foundation for studying the biosynthesis and function of lipid species in health and most prevalent joint diseases.

## Introduction

Synovial fluid (SF) can be viewed as an ultrafiltrate of plasma that contains locally synthesized factors, such as cytokines; growth factors; and lubricating compounds, such as lubricin, hyaluronic acid, and phospholipids. The chief functions of SF are shock absorption; load bearing; lubrication of articular surfaces, such as those of cartilage, the meniscus, tendons, and ligaments; and nutrition and communication medium of joints.

Altered composition or concentrations of SF components are linked to osteoarthritis (OA) and rheumatoid arthritis (RA) [1]–[3]. A precise profile of the chemical composition of SF during disease-related alterations will increase our knowledge about the pathogenesis and possible options to treat these joint diseases.

Sphingolipids (SLs) are a class of lipids that include ceramide (Cer) species, sphingomyelins (SMs) and more complex glycosphingolipids. A common constituent of all SLs is the sphingoid base, which is an organic aliphatic amino alcohol sphingosine (SPH) or a structurally similar compound [4]. The metabolic pathways of SLs form complex networks of reactions that involve many enzymes and intermediate metabolites that are needed for the biosynthesis, degradation, and remodeling of individual SLs [4]–[6].

SLs are structural components of plasma membranes and bioactive molecules that have significant functions in proliferation and growth as well as differentiation, cellular signal transduction, and apoptosis in many mammalian cells for instance fibroblast-like synoviocytes (FLSs) and neural cells [7]–[11]. The function of SLs depends on their acyl chain length and degree of saturation.

The balance between levels of individual SLs is critical. For instance, sphingosine-1-phosphate (S1P) antagonizes Cer-mediated apoptosis [9]. Exogenous Cer in turn was reported to regulate proliferation in human FLSs from OA and RA patients [7]. Treatment of FLSs with micromolar concentrations of C6-Cer inhibits proliferation through G_o_/G_1_ arrest, similar to what has been observed after serum starvation [7].

Cardiolipins (CLs) are tetra-acylated glycerophospholipids that are part of the inner mitochondrial membranes in eukaryotic cells. CLs regulate energy production [12], [13], whereas free radical oxidation products of CLs are important mediators of mitochondria-dependent apoptosis [14]–[16]. Altered levels and composition of CLs are linked to diseases, such as diabetes [13], heart failure [13], [17], Parkinson disease [18], and a rare cardiomyopathy, known as Barth syndrome [19].

Bis(monoacylglycero)phosphate (BMP) is an acidic phospholipid and an isomer of phosphatidylglycerol (PG). BMPs are formed on the surface of intralysosomal vesicles during the degradation of PGs and CLs and stimulate the enzymatic hydrolysis of membrane-bound SLs. The highest levels of negatively charged BMPs are found in the internal vesicles of lysosomes, and the concentration of BMPs increases as late endosomes convert into lysosomes [20]. BMPs are important for endosomal and lysosomal function, and altered levels of BMP species have been reported in a group of diseases, known as lysosomal storage disorders, in which lysosomal dysfunction leads to the accumulation of secondary metabolites, as observed in Gaucher disease, Fabry disease, mucopolysaccharidosis, Pompe disease, and drug-induced phospholipidosis [20], [21].

Phosphatidic acid (PA) is a biosynthetic precursor of lipids, like PGs, CLs, and BMPs. There are two lipids that are chemically similar to PA: lysophosphatidic acid (LPA) and cyclic PA (cPA). LPA has an identical chemical structure to PA, except that it contains a single fatty acid (FA). LPA regulates many physiological and pathophysiological responses and is one of the most active naturally occurring lipids [22]. cPA act as a lipid mediator with several biological activities. Notably, cPA was recently reported to possess some antiinflammatory and chondroprotective activities in a rabbit model of OA [23].

The normal use of articular joints may damage cells found within SF which may be thus an additional source of many components of SF, including intracellular lipids that are not actively secreted into the SF. Chondrocytes, synovial lining cells, leukocytes (predominantly T cells), and cells from the meniscus, tendon, and ligaments are normally found at the borderline of SF or in SF. In addition, SF is an ultrafiltrate of the plasma so that lipids might also derive from the blood.

The levels, composition, and functions of SL, CL, LPA, PA, BMP, PG and lysophosphatidylglycerol (LPG) species in SF are unknown. Thus, we aimed to identify and quantify SL, CL, LPA, PA, LPG, PG, and BMP species. In particular, we wanted to investigate whether these lipid species are related to the health status of synovial joints using SF from normal donors as well as from patients with RA, early (eOA) or with late stages (lOA) of OA.

## Materials and Methods

### Inclusion and Exclusion Criteria

The SF originated from 9 deceased donors, 18 patients with RA and 30 patients with OA (Table 1). The present study was approved by the Ethical Review Committee of the Faculty of Medicine (Justus-Liebig-University of Giessen, Germany), and all patients provided written informed consent to donor samples for research. The Ethical Review Committee (protocol #62/06) waived the need for consent to be obtained from relatives of deceased donors since a judicial order to perform autopsy existed and, furthermore, to avoid additional emotional drain on relatives.

**Table 1:** Characteristics of synovial fluid donors.

	Postmortem donors. n = 9	Patients with eOA. n = 17	Patients with lOA. n = 13	Patients with RA. n = 18
**Age**	22 (21–25)	36 (25–49)	70 (67–74)	64 (49–71)
**female/male**	1/8	6/11	7/6	14/4
**BMI**	24.8 (20.8–25.2)	24.7 (22.6–26.9)	27.7 (25.8–30.1)	29.5 (24.8–32.8)
**CRP**	nd	0.5 (0.5–1.0)	1.6 (1.13–1.85)	13.2 (5.3–38.8)
**No. of cells/µl SF**	nd	nd	nd	6100 (2588–12,925)
**DAS28**	nd	nd	nd	3.48 (3.32–4.56)

Inclusion criteria: both genders, age 18–85 years inclusive, BMI<40, CRP≤3 mg/L, and all CRP levels for RA. Exclusion criteria: joint infection; severe liver or kidney disease; any surgery within the last 3 months; knee joint surgery within the last 6 months; diabetes mellitus (OA); drug abuse; intraarticular treatment with hyaluronate; or corticosteroid treatment within the last 3 months; HIV infection; tumor/cancer.

**Figure 1:**
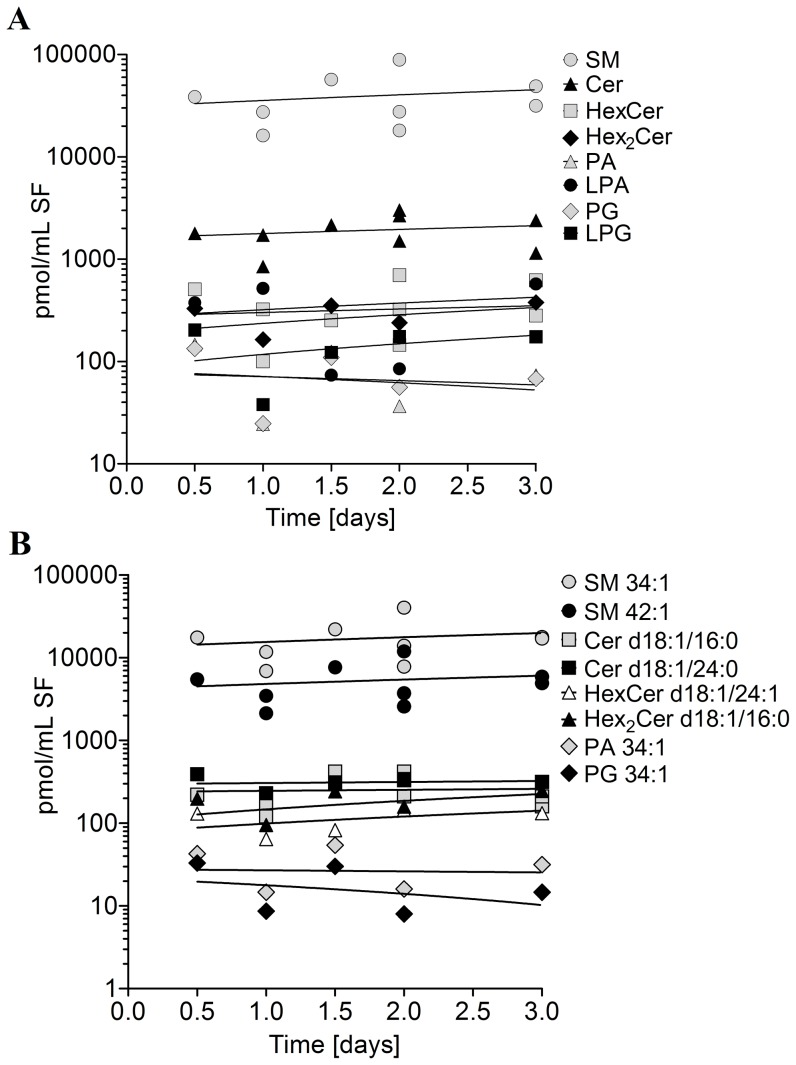
Postmortem stability of lipids extracted from human synovial fluid of healthy knee joints used as controls. Lipids were determined by electrospray ionization tandem mass spectrometry (ESI-MS/MS) or liquid chromatography coupled with tandem mass spectrometry (LC-MS/MS) as outlined in *Material and Methods*. Values are displayed as a scatterplot of the concentration of each lipid class and species by postmortem time. (**A**): Lipid classes, (**B**): Lipid species. SM-sphingomyelin, Cer-ceramide, HexCer-hexosylceramide (most likely glucosylceramide), Hex_2_Cer-dihexosylceramide (most likely lactosylceramide), PA-phospatidic acid, LPA-lysophosphatidic acid, PG-phosphatidylglycerol, LPG-lysophosphatidylglycerol.

We used the Outerbridge classification scale (OU) to subcategorise the osteoarthritic changes in the joints as early (eOA) and late (lOA) stages of the disease [24]. For this, the 6 cartilage surfaces of the patella, trochlea and the medial and lateral sides of the femoral condyles and the tibia were macroscopically evaluated and the resulting cumulative OU score was divided by six in order to obtain an average score valid for the entire joint. Early osteoarthritic disease stages were defined as those which showed an average score ≤2, while late stages of the disease were defined as those with an OU score >2. Patients with RA were classified as described in the guidelines of the American College of Rheumatology [25]. SF was removed during autopsies of knee healthy donors at the Institute of Forensic Medicine, University of Giessen at the times after death described in Figure 1.

The SF was withdrawn into a syringe either during an arthroscopy (eOA) or during an otherwise necessary knee puncture (RA). In contrast, the SF of patients with late stage disease (lOA) was obtained during a knee prosthetic implantation.

### Processing of the Synovial Fluid

The SF was processed as described elsewhere [26]. Briefly, SF was macroscopically evaluated, pressed through a 1.2 micron filter, and then 10% (v/v) inhibitors were added in order to inhibit proteases and phospholipases. This was followed by centrifugation of the SF for 45 min at 16,100×g and RT in order to pellet the cellular debris [26], [27]. Finally, the supernatant was frozen at −86°C for further lipid analysis.

### Extraction of Sphingolipids and Phospholipids

The cell and cellular debris-free supernatant was extracted in the presence of internal lipid standards (Avanti Polar Lipids, Alabaster, AL, USA) in order to specifically quantify extracellular SMs and Cer species [26], [28]. In addition, aliquots of the SF supernatant were extracted with butanol in order to determine hexosylceramide (HexCer) species, dihexosylceramide (Hex_2_Cer) species, sphingoid bases, sphingosylphosphorylcholine (SPC) [29], S1P, LPAs, CLs, BMPs, PGs, and PAs [30], [31]. In brief, SF was first buffered with 400 µl 30 mM citric acid and 40 mM disodium hydrogen phosphate. Lipids were then extracted with 1-butanol, dried, and redissolved in 50 µl ethanol.

### Mass Spectrometric Quantification of Lipid Species

As already described the SM and Cer species were determined using electrospray ionisation mass spectrometry (ESI-MS/MS) [28], [32]. In addition, we used MS/MS after liquid chromatographic separation (LC-MS/MS) to determine HexCer species, Hex_2_Cer species, sphingoid bases, SPC [29], S1P, LPAs [30], CLs, BMPs, PGs and PAs [31]. Lipid species were annotated as published [33].

### Data Analysis

A possible dilution of SF by a joint effusion would result in a clearly reduced concentration of certain lipids as determined by ESI-MS/MS. The liver produces urea which is not synthesized or metabolized in articular joints. Urea diffuses without any restriction across the synovium and can therefore be used to evaluate unknown dilution. The dilution effect was corrected by the method described by Kraus *et al* [26], [34]. For this purpose, the concentration of urea was first measured in both SF and serum so that a dilution factor for the SF according to Kraus *et al* [34] could be calculated. Urea was determined using a commercially available kit (BioAssay Systems, Hayward, CA, USA).

### Statistical Analysis of the Measured Values Obtained

In our exploratory study, a non-Gaussian distribution of the measured values was assumed so that a non-parametric statistical analysis was carried out. The Kruskal-Wallis test was used to identify statistically significant differences in the levels of lipid species between different cohorts. The false discovery rate was controlled at 5% (Benjamini-Hochberg adjustment).. For the selected species, the Steel-Dwass multiple comparisons was applied to determine statistically significant differences in lipid concentrations between individual cohorts (control, eOA, lOA, RA) while controlling the family-wise error-rate at 5%.

We used the statistical software “R” version 2.14.0 to perform the Kruskal-Wallis test, Benjamini-Hochberg adjustment as well as the Steel-Dwass tests. The data obtained are presented as medians with interquartile ranges for the box plots. The values quoted in the text represent the median together with the interquartile range in brackets.

## Results

### Sphingomyelin Molecular Species

**Figure 2:**
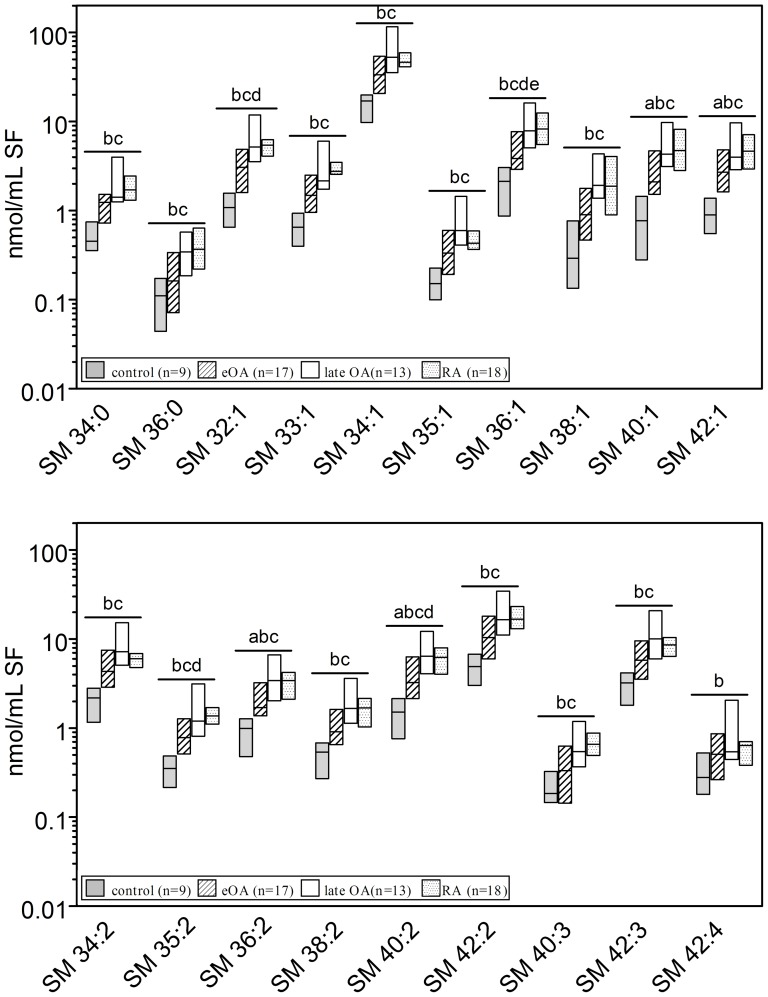
Concentrations of sphingomyelin species in human knee synovial fluid. Synovial fluid was obtained from donors serving as controls and from patients with early osteoarthritis (eOA), late OA (lOA), and rheumatoid arthritis (RA). SM species were determined by electrospray ionization tandem mass spectrometry as outlined in *Material and Methods*. Species annotation is based on the notion that 2 hydroxyl groups are linked to a sphingoid base. Values are presented as median and interquartile range. Significance was considered in the following way: a: p≤0.05: control vs eOA; b: p≤0.05: control vs lOA; c: p≤0.05: control vs RA; d: p≤0.05: eOA vs lOA; and e: p≤0.05: eOA vs RA. SM- sphingomyelin.

Using established ESI-MS/MS method the changes in the concentrations of SM species were examined (28). Of the SL classes that we examined, SMs were the most abundant in SF. The concentration of total SMs in control SF was 39 nmol/ml, multiplying 2.4-fold in eOA (92 nmol/ml; p<0.05), 4.4-fold in lOA (172 nmol/ml; p≤0.001), and 3.2-fold in RA (126 nmol/ml; p≤0.001). Nineteen SM species, based on the length and saturation of the attached FAs, were identified in human SF as shown in Figure 2. SM 34:1 was the predominant species among SMs, accounting for 38% to 44% of total SMs in all cohorts (Figure 2). In addition, versus control SF, 19 SM species were elevated by 2.4-fold (2.2–2.8-fold; p<0.05) in eOA, 4.8fold (4.1–5.4-fold; p≤0.001) in lOA, and in RA SF 3.5-fold (2.8–4.2-fold; p≤0.001). Notably, all SM species had risen approximately 2-fold in SF from eOA to lOA (p≤0.05, Figure 2).

### Ceramide Molecular Species

**Figure 3:**
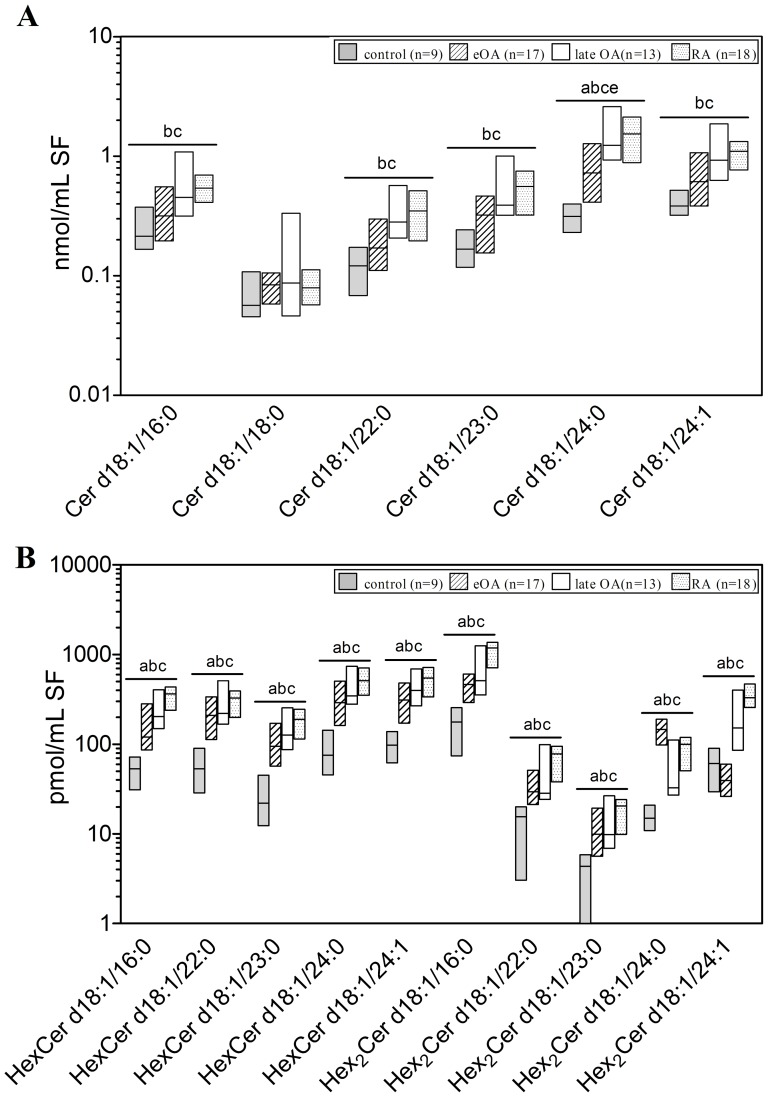
Concentrations of ceramide species in human knee synovial fluid. Synovial fluid was obtained from donors serving as controls and from patients with early osteoarthritis (eOA), late OA (lOA), and rheumatoid arthritis (RA). Cer species were determined by electrospray ionization tandem mass spectrometry as outlined in *Material and Methods*. Values are presented as median and interquartile range. Significance was considered in the following way: a: p≤0.05: control vs eOA; b: p≤0.05: control vs lOA; c: p≤0.05: control vs RA; d: p≤0.05: eOA vs lOA; and e: p≤0.05: eOA vs RA. (**A**): Cer species, (**B**): HexCer and Hex_2_Cer species. Cer- ceramide, HexCer- hexosylceramide (most likely glucosylceramide), Hex_2_Cer- dihexosylceramide (most likely lactosylceramide).

In order to quantify Cer species, ESI-MS/MS analysis was used. Furthermore, LC-MS/MS method was performed to quantify HexCer and Hex_2_Cer species. Within analysed SLs, the second most prominent group of SLs in human SF was Cer species. The concentration of total Cer species was 1.4 nmol/ml in SF from controls, elevated 2-fold in SF from eOA (2.8 nmol/ml; n.s.), 3.9-fold in lOA SF (5.5 nmol/ml; p≤0.01), and 3-fold in SF from RA (4.2 nmol/ml; p≤0.001). Six Cer species were identified; Cer d18:0/24:0 was the predominant species (Figure 3A). As expected, the level of most Cer species was higher in lOA and RA SF, such as Cer d18:1/16:0, Cer d18:1/22:0, Cer d18:1/23:0, and Cer d18:1/24:1. Notably, most Cer species contained saturated FAs, constituting 68.9% to 74.9% of total Cer species in all cohorts (Figure 3A).

**Figure 4:**
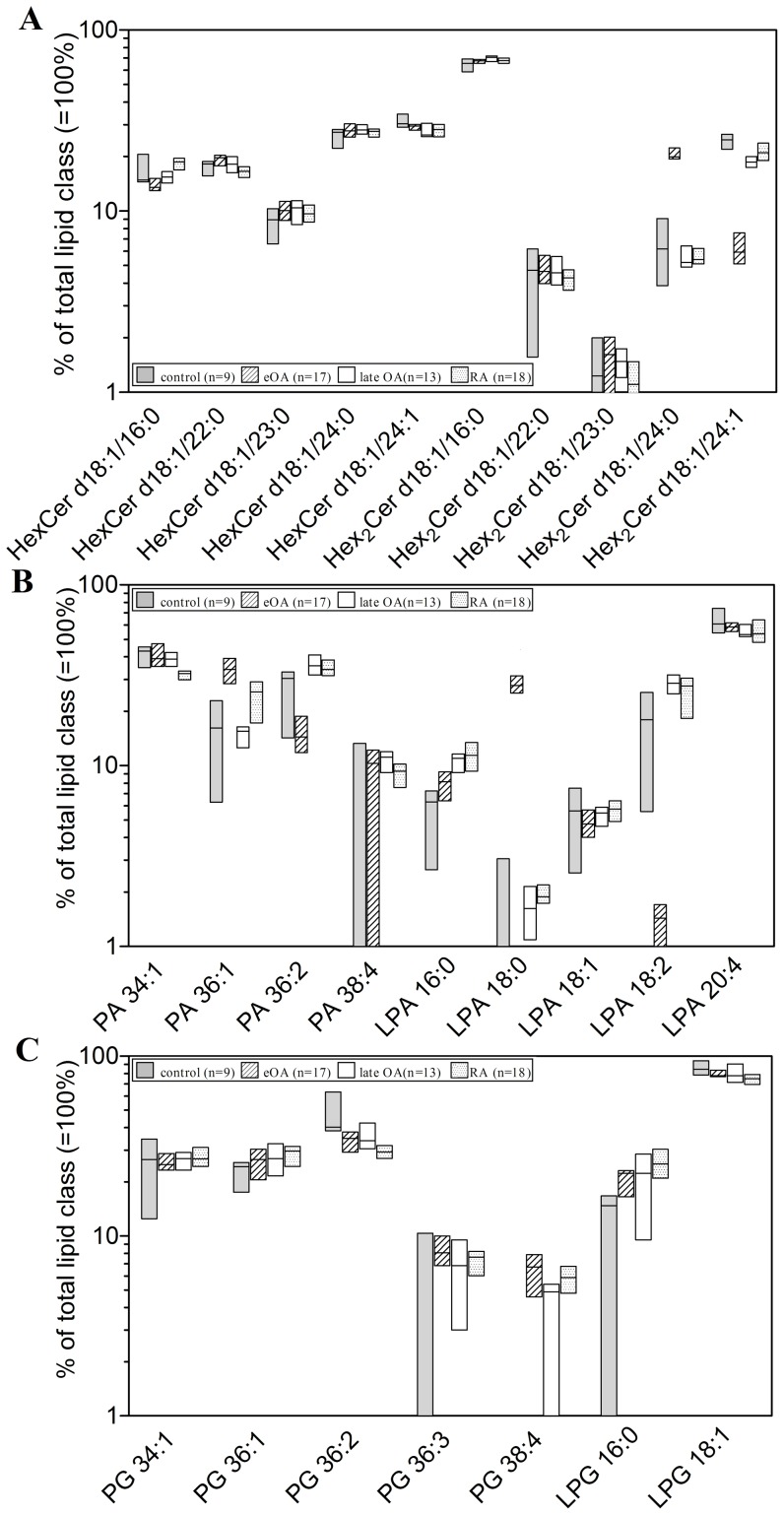
Relative distribution of HexCer and Hex_2_Cer, PA, LPA, PG and LPG species in in human knee synovial fluid. Synovial fluid was obtained from donors serving as controls and from patients with early osteoarthritis (eOA), late OA (lOA), and rheumatoid arthritis (RA). The data show the percentage of the lipid species from the total corresponding lipid class ( = 100%). Values are presented as median and interquartile range. (**A**): HexCer and Hex_2_Cer species, (**B**): PA and LPA species, (**C**): PG and LPG species. HexCer- hexosylceramide (most likely glucosylceramide), Hex_2_Cer- dihexosylceramide (most likely lactosylceramide), PA- phosphatidic acid, LPA- lysophosphatidic acid, PG- phosphatidylglycerol, LPG- lysophosphatidylglycerol.

Further, the molecular species of HexCer and Hex_2_Cer were quantified. Five HexCer species and 5 Hex_2_Cer species were detected in human SF (Figure 3B and 4A). The percentage distribution of these species differed slightly between all cohorts, except for Hex_2_Cer d18:1/24:0 which was high in eOA, and Hex_2_Cer d18:1/24:1, which was low in eOA (Figure 4A). HexCer species and Hex_2_Cer species were low in control SF but higher in the SF of all other cohorts. In comparison with control SF, the concentrations of HexCer species increased by 3.9-fold (3.4–4.2-fold; p≤0.001) in eOA, 5.8-fold (4.8–6.1-fold; p≤0.01) in lOA, and in RA SF 5.8-fold (5.3–6.1-fold; p≤0.001) (Figure 3B), whereas Hex_2_Cer species rose by 3.4-fold (3.2–5.5-fold; p≤0.001) in eOA, 4.5-fold (4.4–5.2-fold; p≤0.05) in lOA, and in RA SF 6.9–fold (6.9–7.3-fold; p≤0.001) (Figure 3B).

### Lysosphingolipids and Their Phosphates

The values for S1P and sphinganine-1-phosphate in SF were below the limit of detection (6 pmol/ml for undiluted samples). We identified the following species in RA SF: SPH d18:0 [11.3 pmol/ml (0.0–21.2 pmol/ml)], SPH d18:1 [20.0 pmol/ml (15.2–24.8 pmol/ml)], SPC d18:0 [1.0 pmol/ml (0.0–1.8 pmol/ml)], and SPC d18:1 [16.1 pmol/ml (10.8–31.2 pmol/ml)]. Only 2 SL species in OA SF [SPH d18:1, 5.2 pmol/ml (0.0–16.9 pmol/ml) and SPC d18:1, 10.1 pmol/ml (4.6–19.5 pmol/ml)] were detected.

### Molecular Species of Cardiolipins and BMPs

CLs possess a unique dimeric structure, bearing phosphatidyl moieties that are linked by a bridging glycerol, to which 4 FAs are attached. Our mass spectrometric analysis only enabled us to measure the sum of double bounds and carbon atoms in each of the 2 phosphatidyl moieties. The CL species concentrations were below the limit of detection, except for CL 36:4/36:4, which was elevated in RA SF.

Only 2 BMP species (BMP 18:1/18:1 and BMP 18:2/18:1) were above the limit of detection (Table 2). The highest levels of these species were observed in RA SF. In comparison with control SF, the levels of these BMP species rose by 1.6-fold (1.4–1.7-fold; ns) in all other cohorts.

**Table 2:** Concentrations of bis(monoacylglycero)phosphate (BMP) species.

Concentration [pmol/ml]				
BMP-specie	control (n = 9)	eOA (n = 17)	lOA (n = 13)	RA (n = 18)
BMP 18:1/18:1	27.8 (17.5–46.8)	23.1 (15.4–37.9)	36.7 (22.9–74.7)	24.2 (19.8–30.9)
BMP 18:2/18:1	15.6 (9.27–20.0)	16.8 (11.5–25.5)	25.4 (15.9–46.3)	18.8 (13.9–23.6)

Concentrations of bis(monoacylglycero)phosphate (BMP) species in knee synovial fluid obtained from donors serving as controls and from patients with early osteoarthritis (eOA), late OA (lOA), and rheumatoid arthritis (RA). BMP species were determined by electrospray ionization tandem mass spectrometry as outlined in *Material and Methods*. Values are presented as median and interquartile range in the brackets. BMP-Bis(monoacylglycero)phosphate, eOA-early osteoarthritis, lOA-late osteoarthritis, RA-rheumatoid arthritis.

### Molecular Species of PA, PG, and Lysophospholipids (LPA, LPG)

**Figure 5:**
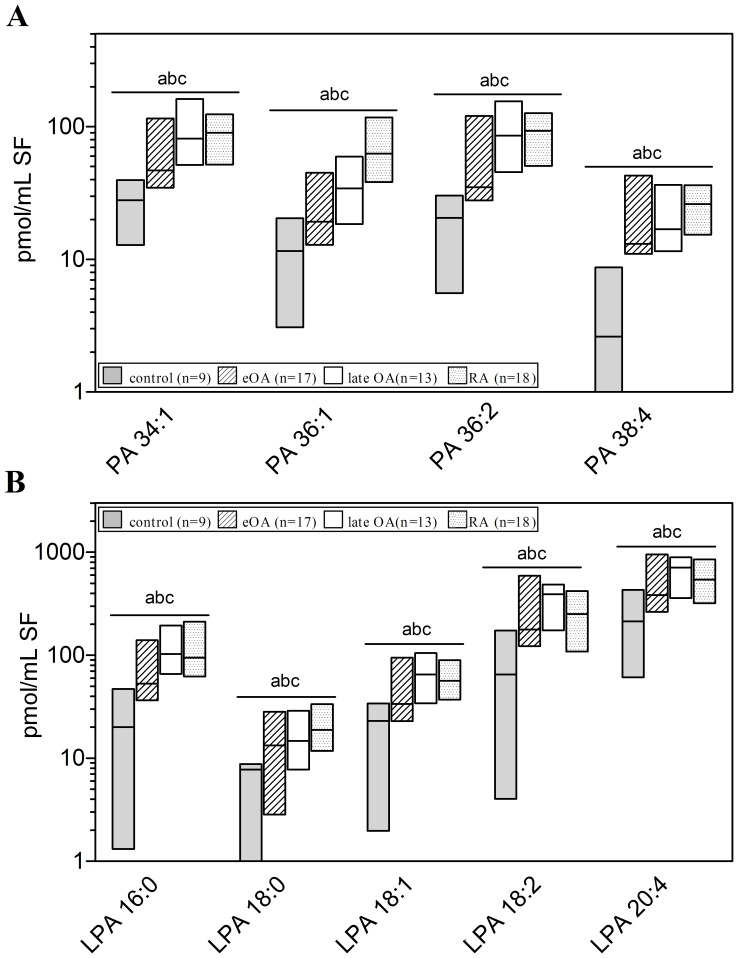
Concentrations of phosphatidic acid (PA) and lysophosphatidic acid (LPA) species in human knee synovial fluid. Synovial fluid was obtained from donors serving as controls and from patients with early osteoarthritis (eOA), late OA (lOA), and rheumatoid arthritis (RA). PA and LPA species were determined liquid chromatography coupled with tandem mass spectrometry as outlined in *Material and Methods*. Species annotation is based on the notion that only ester bonds are present. Values are presented as median and interquartile range. Significance was considered in the following way: a: p≤0.05: control vs eOA; b: p≤0.05: control vs lOA; and c: p≤0.05: control vs RA. (**A**): PA species, (**B**): LPA species. PA- phosphatidic acid, LPA- lysophosphatidic acid.

**Figure 6:**
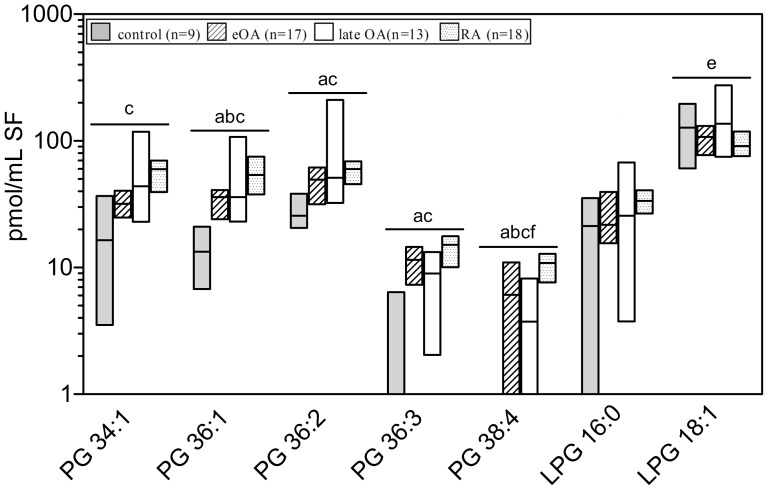
Concentrations of phosphatidylglycerol (PG) and lysophosphatidylglycerol (LPG) species in human knee synovial fluid. Synovial fluid was obtained from donors serving as controls and from patients with early osteoarthritis (eOA), late OA (lOA), and rheumatoid arthritis (RA). PG and LPG species were determined by liquid chromatography coupled with tandem mass spectrometry as outlined in *Material and Methods*. Species annotation is based on the notion that only ester bonds are present. Values are presented as median and interquartile range. Significance was considered in the following way: a: p≤0.05: control vs eOA; b: p≤0.05: control vs lOA; c: p≤0.05: control vs RA; d: p≤0.05: eOA vs lOA; e: p≤0.05: eOA vs RA; and f: p≤0.05: lOA vs RA. PG- phosphatidylglycerol, LPG- lysophosphatidylglycerol.

As a class, PAs and PGs contain 2 FA chains. Our mass spectrometric analysis allowed us only to determine the sum of double bonds as well as carbon atoms in the FAs, as shown in Figure 5A and 6.

Four PA and 5 LPA species, based on the number of double bounds and chain length, were detected in human SF (Figure 4B and 5). The relative distribution of these species was similar in all cohorts, except for high levels of LPA 18:0 in eOA and low levels of LPA 18:2 in eOA (Figure 4B). Notably, the concentrations of all PA and LPA species were similar in eOA, lOA, and RA SF. However, in comparison with control SF, the levels of all PA species increased 3.0-fold (2.3–3.8-fold; p≤0.05) in eOA, 4.5-fold (3.9–4.9; p≤0.05) in lOA, and in RA SF 6.7-fold (6.0–7.5; p≤0.001) (Figure 5A). Similarly, versus control SF, the levels of all LPA species rose by 2.7-fold (2.4–3.2-fold; p≤0.01) in eOA, 4.0-fold (3.6–4.4; p≤0.01) in lOA, and in RA SF 3.6-fold (3.2–4.1; p≤0.001) (Figure 5B).

The relative distribution of PG and LPG species did not differ between cohorts (Figure 5C). The major PG species in human SF were those with 34 and 36 C-atoms (PG 34:1, PG 36:1, and PG 36:2 with concentrations between 0.062 and 3.07 pmol/ml; Figure 6). Five PG and 2 LPG (LPG 16:0 and LPG 18:1) species were found in human SF at concentrations of 0.1–5.0 pmol/ml (Figure 6). Notably, in comparison with control SF, the levels of all PG and LPG species were elevated by 3.5-fold (2.5–5.6-fold; p≤0.01) in eOA, 3.6-fold (2.8–4.6; p≤0.05) in lOA, and in RA SF 2.9-fold (1.9–4.5; p≤0.01). However, the levels of PG and LPG species did not differ between eOA and lOA SF.

### Post Mortem Stability of Sphingolipids in SF

The 9 postmortem donors died of intoxication (4×), multiple trauma (2×), cardiomyopathy (1×), pulmonary embolism (1×), and craniocerebral trauma (1×). The SF was obtained with a syringe during the autopsy of 9 adult donors without any known joint disease within a window of several hours to up to 5 days after the time-point of death. Since the stability of sphingolipids in SF as a function of the post-mortem time after time of death is unknown, the concentrations of several lipid species that exist at high levels in the SF (SM 34:1, SM 42:2, Cer d18: 1/24: 0) were plotted against the time between death and autopsy. Figure 6 show that no relevant changes occur in the level of sphingolipids within the time frame of sample collection. Furthermore, no discordant values is obvious in Figure 1 indicating that the cause of death appears to have no impact on the level of lipid species. However, the numbers of investigated SF per cause of death is too low to generalize this observation.

In order to confirm the stability of the lipid species mathematically, a linear regression of the data was obtained, and the slope of the line was calculated together with the 95% confidence interval. None of the slopes differed from 0 (values not shown).

### Sphingolipids and Age of Donors

**Figure 7:**
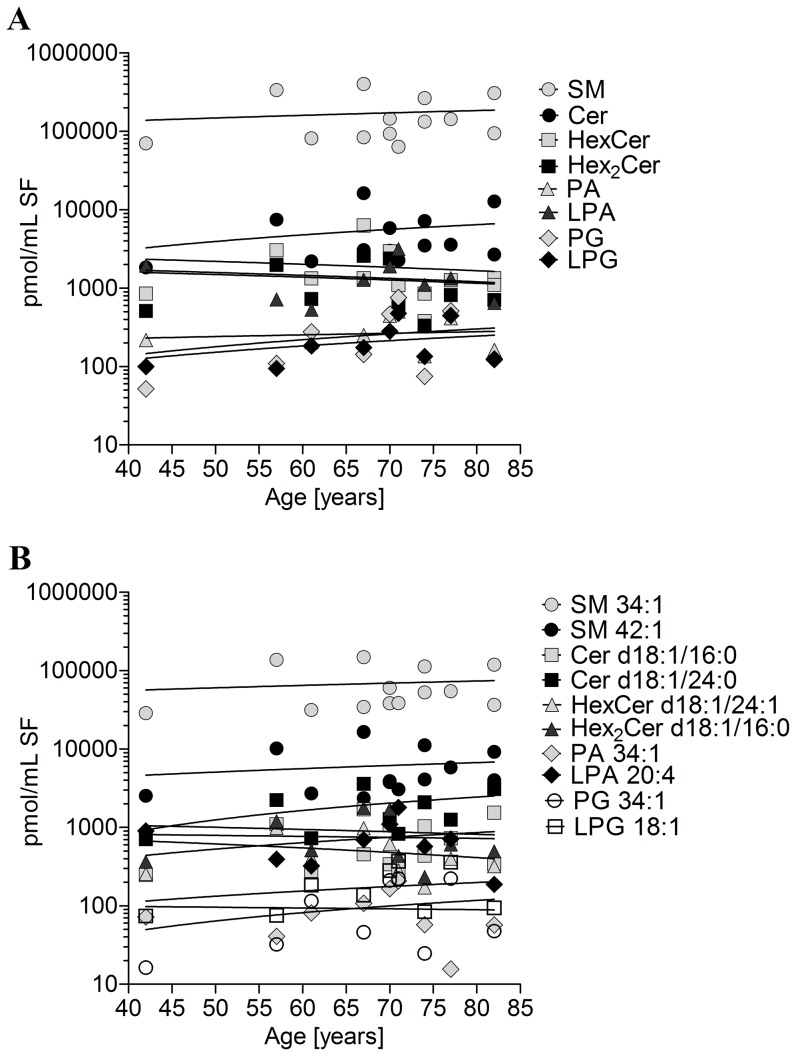
Concentrations of lipids in human synovial fluid as a function of the age of patients with late stage osteoarthritis. Lipids were determined by electrospray ionization tandem mass spectrometry (ESI-MS/MS) or liquid chromatography coupled with tandem mass spectrometry (LC-MS/MS) as outlined in *Material and Methods*. Values are displayed as a scatterplot of the concentration of each lipid class and species by age of donors. (**A**): Lipid classes, (**B**): Lipid species. SM-sphingomyelin, Cer-ceramide, HexCer-hexosylceramide (most likely glucosylceramide), Hex_2_Cer-dihexosylceramide (most likely lactosylceramide), PA-phospatidic acid, LPA-lysophosphatidic acid, PG-phosphatidylglycerol, LPG-lysophosphatidylglycerol.

It is not known whether the level of sphingolipids in SF may be also dependent on the age of donors. Furthermore, the four cohorts of our study differ from each other with respect of their average age. Therefore the concentration of investigated lipid classes and species was plotted against the age of each donor within each cohort. Figure 7 as well as Figures S1, S2, S3 shows that the age of donors had no impact on the concentrations of lipids within the four investigated cohorts. Also, a linear regression of the data was calculated together with the slope and the 95% confidence interval revealing, that none of the slopes differed significantly from 0 (values not shown).

### Adjustment of Data

The adjusted values were obtained from there corresponding nonadjusted concentrations by multiplying with a dilution factor [34] and resulting data are shown both for classes (Table 3) and individual species (Table S1). During OA water SF might be diluted by water due to inflammation-induced effusion. Therefore, the concentrations of lipid species were corrected for possible dilution. The concentrations of urea were determined within serum and SF in order to calculate a dilution factor for SF; this procedure was formerly developed to adjust for the dilution introduced by lavage during some biomarker studies. Compared with the level of lipids in SF of controls, eOA, lOA, and RA had elevated concentrations of most lipid species independent of whether they were adjusted or nonadjusted. However, the differences between most lipid species in eOA and lOA were more obvious using the adjusted values (Table S1)

**Table 3:** Impact of dilution factor on the concentrations of lipid classes.

Lipid class	eOA (n = 17) [%]	lOA (n = 13) [%]	RA (n = 18) [%]
**SM**	118.2 (110.5–123.8)	157.1 (146.5–173.5)	88.1 (83.4–89.2)
**Cer**	130.7 (122.6–132.9)	157.7 (154.9–164.1)	81.2 (78.4–87.3)
**HexCer**	110.1 (110.1–111.6)	153.1 (152.7–158.6)	86.0 (82.8–88.5)
**Hex_2_Cer**	103.9 (99.8–105.4)	138.4 (135.5–139.8)	81.2 (77.5–81.9)
**PA**	103.3 (98.9–106.8)	180.0 (165.7–185.7)	82.8 (79.1–85.6)
**LPA**	109.9 (108.3–113.3)	176.3 (152.6–190.6)	84.2 (80.1–88.3)
**PG**	100.0 (98.9–108.5)	123.9 (121.1–126.4)	84.2 (80.8–86.4)
**LPG**	107.6 (101.9–113.3)	143.2 (133.0–153.4)	82.9 (81.9–83.9)
**BMP**	97.3 (95.4–99.3)	165.0 (165.0–165.0)	79.6 (77.9–81.2)

Impact of dilution factor on the concentrations of lipid classes as expressed as percentage of uncorrected values ( = 100%). Lipids were determined by electrospray ionization tandem mass spectrometry or liquid chromatography coupled with tandem mass spectrometry, and corrected for possible dilution as outlined in *Material and Methods*. Values are median and interquartile range. eOA-early osteoarthritis, lOA-late osteoarthritis, RA-rheumatoid arthritis, SM-sphingomyelin, Cer-ceramide, HexCer-hexosylceramide (most likely glucosylceramide), Hex_2_Cer-dihexosylceramide (most likely lactosylceramide), PA-phosphatidic acid, LPA-lysophosphatidic acid, PG-phosphatidylglycerol, LPG-lysophosphatidylglycerol, BMP-bis(monoglycero)phosphate.

## Discussion

Lipids such as SLs, PAs, LPAs, LPGs, and BMPs and other groups of lipids are known to be bioactive molecules, but their location and function in SF of articular joints remain to be discovered. The lack of knowledge on the profile, quantity, and function of these lipids in human SF underscore the need for detailed studies in this area. Changes in the composition and concentrations of various lipids have been already linked with several human disorders [19], [26].

We have reported that concentrations of phospholipids species increase in SF of RA and OA compared with SF obtained from donors used as controls [26]. The principle finding of our current study is that a broad spectrum of SL species, their precursors, and intermediate metabolites was found in human SF. Moreover, the concentrations of 41 lipids in eOA SF, 48 species in lOA SF, and 50 species in RA SF increase significantly in comparison with control SF. Notably, the levels of 21 lipid species were altered between eOA and lOA SF, indicating that the lipid composition of SF reflects the severity of OA disease. Thus, our findings may be used to develop biomarkers to discriminate eOA from lOA, and eOA from healthy joints.

Until recently, there have been no sensitive methods to detect these species at low concentrations in biological materials. The lipidomic methods that we used in this study [28]–[32], allowed us to identify and quantify many lipid species that have not been reported in SF. This is why our lipidomic investigation reports for the first time about the composition of SL and minor glycerophospholipids species in human SF.

SMs and Cer species regulate many processes, including stress responses, proliferation and differentiation, apoptosis, and senescence [35]–[37]. Using chondrocytes and explants from rabbit articular cartilage, exogenous cell-permeable C2-Cer at concentrations over 25 µM were noted to induce apoptosis, up regulate MMP-1, -3, and -13, and ultimately induce articular cartilage matrix degradation by collagen type II cleavage and proteoglycan loss from cartilage [38]–[41]. Further, Cer species are reported to be mediators that induce proinflammatory cytokine production and possibly apoptosis in cultured human RA FLS [7], [11] as well as cultured chondrocytes [38]. *In vitro* administration of synthetic exogenous C2- or C6-Cer at concentrations below 25 µM did not affect apoptosis but inhibited the proliferation of FLS in OA and RA [7]. However, intraarticular injection of 34.5 µg C2-Cer into the ankle joints of mice increased the population of apoptotic synovial cells [11].

We observed higher concentrations of several Cer species in SF from RA and lOA compared with control SF indicating that Cer species may be involved in the progression of OA and RA. However, the exact function of extracellular SM and Cer species, alone and in combination, in synovial joints is unknown. Further studies are needed to show whether Cer species are synthesized *de novo* or are the result of higher sphingomyelinase activity.

CLs are nearly exclusively a constituent of the mitochondrial inner membrane: extracellular CLs in body fluids have not been reported. We detected CLs only in RA SF. Thus, we speculate that the presence of CLs in SF is a hallmark of RA and that CLs can be used to develop a novel diagnostic tool for RA. However, it is unknown whether CLs in SF have functions. The source of CLs in RA SF is not known and further studies are needed to identify where the CLs come from. One possible source are the cells that are enriched in RA SF compared with OA and control SF and which can be damaged by normal joint movement so that mitochondrial lipids like CLs are released. Although we removed cells and cellular debris from SF by centrifugation and filtration, these techniques are unable to eliminate molecular micelles.

LPAs can promote the proliferation of many cell types. *In vitro* stimulation with 1 or 5 µM exogenous LPA stimulates the proliferation of rat primary chondrocytes [42], [43]. Moreover, the expression of six G-protein-coupled LPA receptors increases in RA FLS. Thus, LPAs appear to function as lipid mediators between cells displaying growth factor-like activities [44]. We noted elevated levels of all PA and LPA species in SF of RA and OA compared with control SF, consistent with previous findings [45]. The higher concentration of LPAs is likely attributed to increased secretion and activity of enzymes for example phospholipase A_2_ [46] and may point to a possible repair response. However, further studies need to evaluate the functions of LPA species as intercellular lipid mediators that induce cell proliferation and have growth factor-like activities in damaged articular cartilage. Recently, cPA was reported to stimulate the production of HA in human OA articular chondrocytes and to provide some anti-inflammatory and chondroprotective activities in a rabbit model of OA [23]. However, our methods applied did not allow us to quantify cPA in SF.

This study is the first to report about PG species in SF. PGs are important components of the pulmonary surfactant system [47]. Thus, we hypothesize that PG species have similar functions in synovial joints. However, our study demonstrates that PG species exist at low concentrations in human SF. Moreover, the PG species in human SF are also found in human plasma; albeit, human plasma appears to have more PG species than SF [20], [48]. In, contrast to human plasma, in which only saturated LPG species (LPG16:0 and LPG 18:0) have been reported [49], human SF contains a high proportion of monounsaturated LPG 18:1. Notably, versus control SF, the concentrations of all PG species were significantly elevated in eOA, lOA, and RA SF. BMP, a structural isomer of PG, was also detected in SF at low levels, and 2 BMP species were identified. However, the function and source of extracellular PG, LPG, and BMP species are unknown.

In conclusion, this lipidomic investigation presents for the first time a comprehensive survey of SLs and minor glycerophospholipids in human SF. Our mass spectrometric analysis of lipids in SF from patients with eOA, lOA, and RA knee joints indicate disease and stage-dependent differences. Certain species of SM and Cer may, at least in part, be involved in the pathogenesis of OA and RA. The paucity of detailed data on the functions of lipid species in RA and OA underscore the necessity for further studies. Our study lays the foundation for addressing specific questions regarding the biosynthesis and function of lipid species in SF.

## Supporting Information

Figure S1 **Concentrations of lipids in human synovial fluid as a function of the age of donors used as controls.** Synovial fluid was obtained post mortem from donors with healthy knee joints. Lipids were determined by electrospray ionization tandem mass spectrometry (ESI-MS/MS) or liquid chromatography coupled with tandem mass spectrometry (LC-MS/MS) as outlined in *Material and Methods*. Values are displayed as a scatterplot of the concentration of each lipid class and species by age of donors. (**A**): Lipid classes, (**B**): Lipid species. SM-sphingomyelin, Cer-ceramide, HexCer-hexosylceramide (most likely glucosylceramide), Hex_2_Cer-dihexosylceramide (most likely lactosylceramide), PA-phospatidic acid, LPA-lysophosphatidic acid, PG-phosphatidylglycerol, LPG-lysophosphatidylglycerol.
(TIF)

Figure S2 **Concentrations of lipids in human synovial fluid as a function of the age of patients with early stage osteoarthritis.** Lipids were determined by electrospray ionization tandem mass spectrometry (ESI-MS/MS) or liquid chromatography coupled with tandem mass spectrometry (LC-MS/MS) as outlined in *Material and Methods*. Values are displayed as a scatterplot of the concentration of each lipid class and species by age of donors. (**A**): Lipid classes, (**B**): Lipid species. SM-sphingomyelin, Cer-ceramide, HexCer-hexosylceramide (most likely glucosylceramide), Hex_2_Cer-dihexosylceramide (most likely lactosylceramide), PA-phospatidic acid, LPA-lysophosphatidic acid, PG-phosphatidylglycerol, LPG-lysophosphatidylglycerol.
(TIF)

Figure S3 **Concentrations of lipids in human synovial fluid as a function of the age of patients with rheumatoid arthritis.** Lipids were determined by electrospray ionization tandem mass spectrometry (ESI-MS/MS) or liquid chromatography coupled with tandem mass spectrometry (LC-MS/MS) as outlined in *Material and Methods*. Values are displayed as a scatterplot of the concentration of each lipid class and species by age of donors. (**A**): Lipid classes, (**B**): Lipid species. SM-sphingomyelin, Cer-ceramide, HexCer-hexosylceramide (most likely glucosylceramide), Hex_2_Cer-dihexosylceramide (most likely lactosylceramide), PA-phospatidic acid, LPA-lysophosphatidic acid, PG-phosphatidylglycerol, LPG-lysophosphatidylglycerol.
(TIF)

Table S1 **Concentrations of lipid species presented in** **Figure 1** **–** **7** **.** Lipids were determined by electrospray ionization tandem mass spectrometry or liquid chromatography coupled with tandem mass spectrometry as outlined in *Material and Methods*. Data (nmol/ml or pmol/ml) obtained were either uncorrected (normal font) or corrected with the dilution factor per Kraus *et al.* (34, bold font). Values are median and interquartile range. The concentrations of urea were determined within serum and SF to calculate a dilution factor for SF; this procedure was formerly developed to adjust for the dilution introduced by lavage during some biomarker studies. eOA-early osteoarthritis, lOA-late osteoarthritis, RA-rheumatoid arthritis, SM-sphingomyelin, Cer-ceramide, HexCer-hexosylceramide (most likely glucosylceramide), Hex_2_Cer-dihexosylceramide (most likely lactosylceramide), PA-phospatidic acid, LPA-lysophosphatidic acid, PG-phosphatidylglycerol, LPG-lysophosphatidylglycerol, SPH-sphingosine, SPC-sphingosylphosphorylcholine, CL-cardiolipin, BMP-bis(monoglycero)phosphate.
(PDF)
