# Supplementary figures and images for: Sphingolipids in Human Synovial Fluid - A Lipidomic Study
Source: PLoS One. 2014 Mar 19;9(3):e91769. doi: 10.1371/journal.pone.0091769 (PMC3960152; doi:10.1371/journal.pone.0091769)

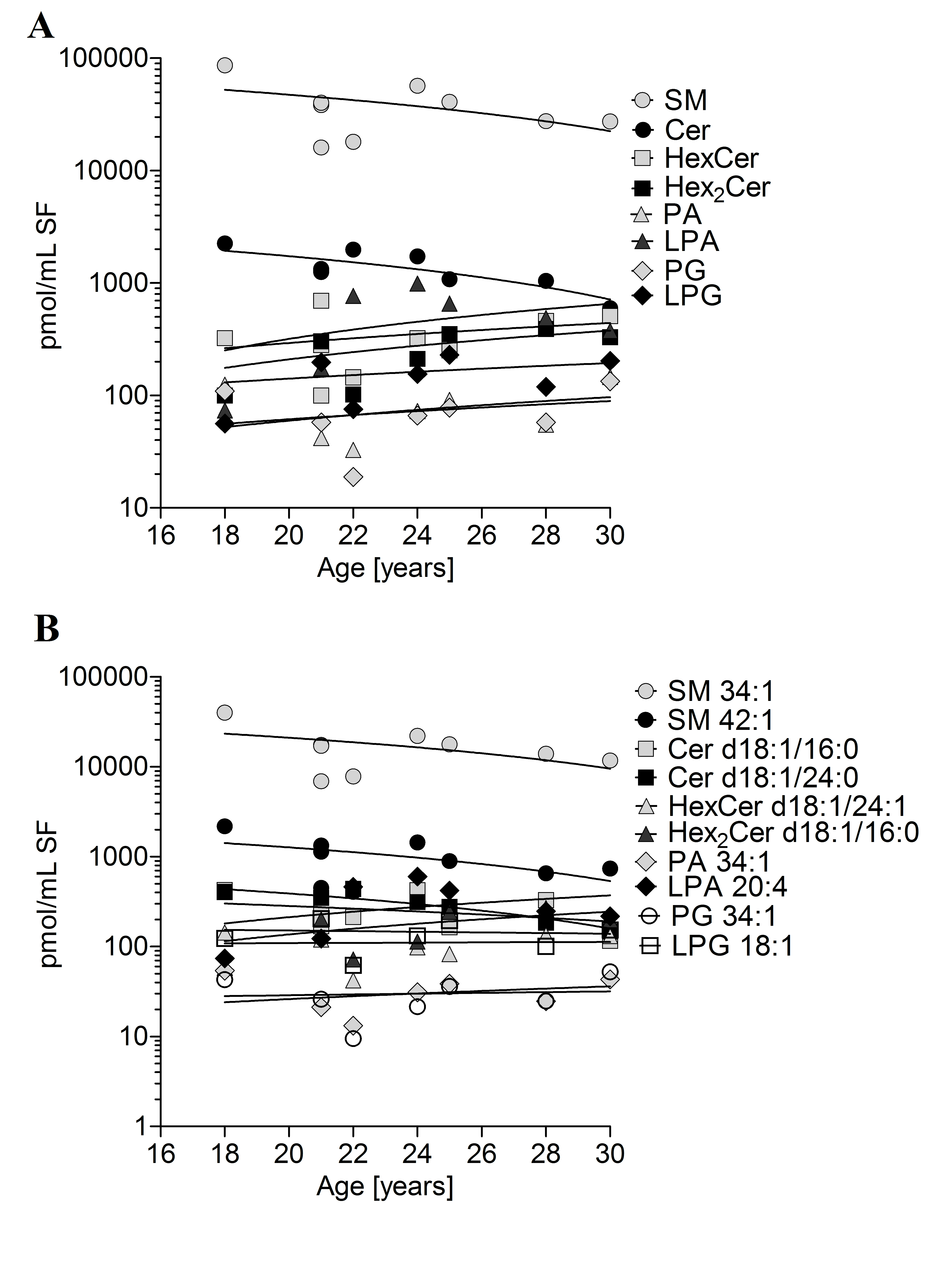

Supplement: Figure S1 — Concentrations of lipids in human synovial fluid as a function of the age of donors used as controls. Synovial fluid was obtained post mortem from donors with healthy knee joints. Lipids were determined by electrospray ionization tandem mass spectrometry (ESI-MS/MS) or liquid chromatography coupled with tandem mass spectrometry (LC-MS/MS) as outlined in Material and Methods . Values are displayed as a scatterplot of the concentration of each lipid class and species by age of donors. (A): Lipid classes, (B): Lipid species. SM-sphingomyelin, Cer-ceramide, HexCer-hexosylceramide (most likely glucosylceramide), Hex2Cer-dihexosylceramide (most likely lactosylceramide), PA-phospatidic acid, LPA-lysophosphatidic acid, PG-phosphatidylglycerol, LPG-lysophosphatidylglycerol. (TIF) [file pone.0091769.s001.tif]

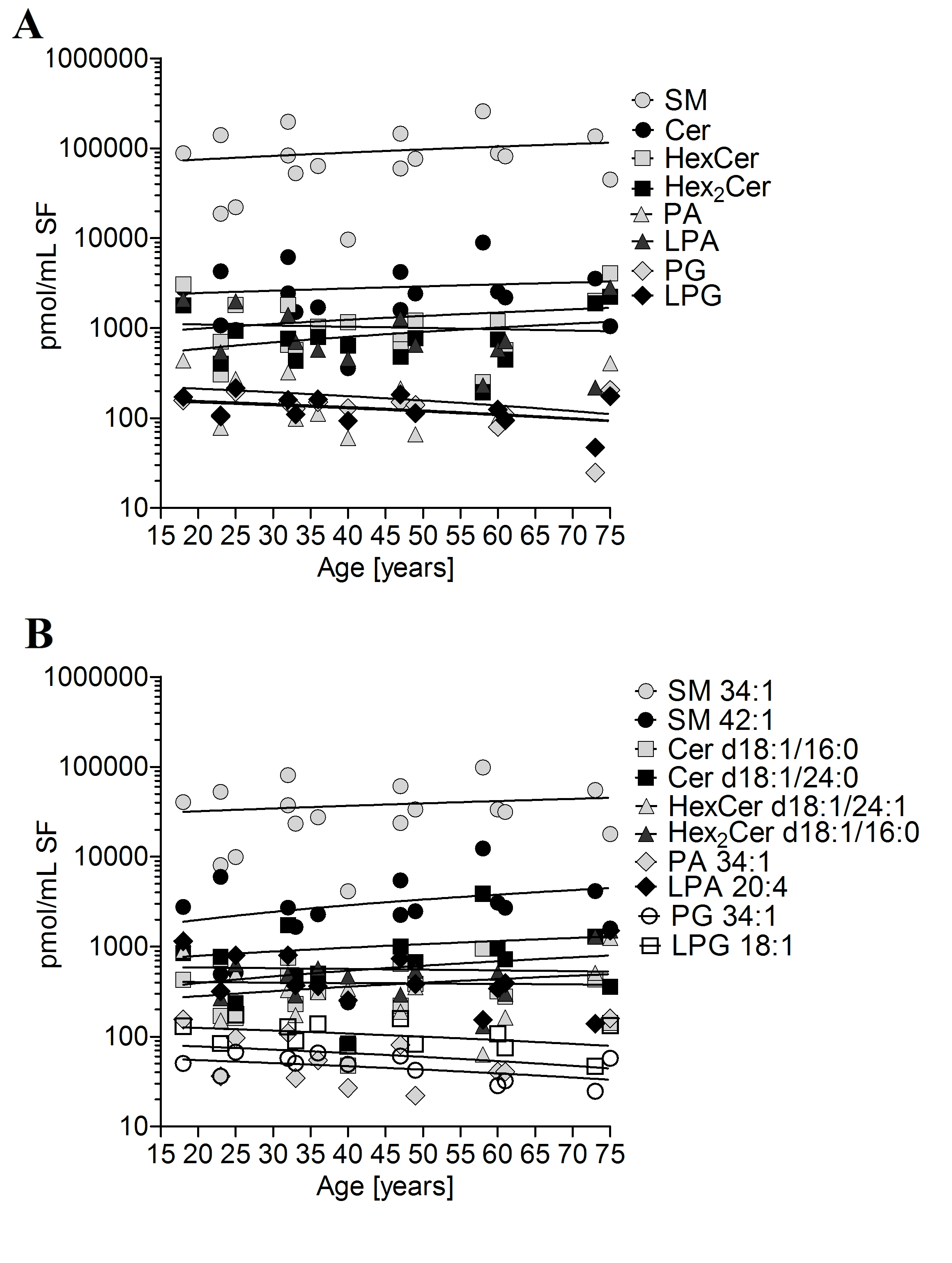

Supplement: Figure S2 — Concentrations of lipids in human synovial fluid as a function of the age of patients with early stage osteoarthritis. Lipids were determined by electrospray ionization tandem mass spectrometry (ESI-MS/MS) or liquid chromatography coupled with tandem mass spectrometry (LC-MS/MS) as outlined in Material and Methods . Values are displayed as a scatterplot of the concentration of each lipid class and species by age of donors. (A): Lipid classes, (B): Lipid species. SM-sphingomyelin, Cer-ceramide, HexCer-hexosylceramide (most likely glucosylceramide), Hex2Cer-dihexosylceramide (most likely lactosylceramide), PA-phospatidic acid, LPA-lysophosphatidic acid, PG-phosphatidylglycerol, LPG-lysophosphatidylglycerol. (TIF) [file pone.0091769.s002.tif]

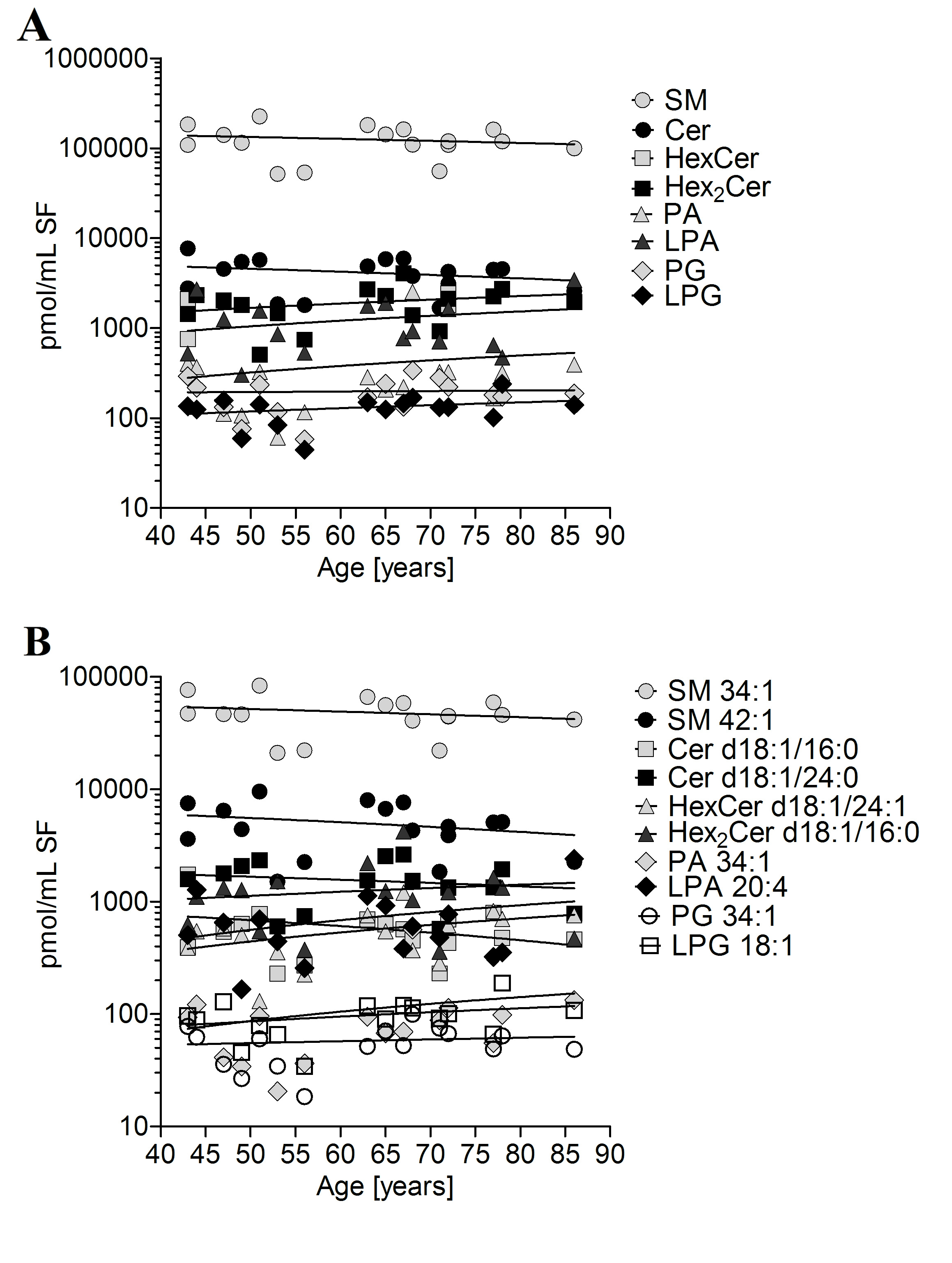

Supplement: Figure S3 — Concentrations of lipids in human synovial fluid as a function of the age of patients with rheumatoid arthritis. Lipids were determined by electrospray ionization tandem mass spectrometry (ESI-MS/MS) or liquid chromatography coupled with tandem mass spectrometry (LC-MS/MS) as outlined in Material and Methods . Values are displayed as a scatterplot of the concentration of each lipid class and species by age of donors. (A): Lipid classes, (B): Lipid species. SM-sphingomyelin, Cer-ceramide, HexCer-hexosylceramide (most likely glucosylceramide), Hex2Cer-dihexosylceramide (most likely lactosylceramide), PA-phospatidic acid, LPA-lysophosphatidic acid, PG-phosphatidylglycerol, LPG-lysophosphatidylglycerol. (TIF) [file pone.0091769.s003.tif]
