# Supplementary material for: Sphingolipids in Human Synovial Fluid - A Lipidomic Study
Source: PLoS One. 2014 Mar 19;9(3):e91769. doi: 10.1371/journal.pone.0091769 (PMC3960152; doi:10.1371/journal.pone.0091769)
Supplement: Table S1 — Concentrations of lipid species presented in Figure 1 – 7 . Lipids were determined by electrospray ionization tandem mass spectrometry or liquid chromatography coupled with tandem mass spectrometry as outlined in Material and Methods . Data (nmol/ml or pmol/ml) obtained were either uncorrected (normal font) or corrected with the dilution factor per Kraus et al. (34, bold font). Values are median and interquartile range. The concentrations of urea were determined within serum and SF to calculate a dilution factor for SF; this procedure was formerly developed to adjust for the dilution introduced by lavage during some biomarker studies. eOA-early osteoarthritis, lOA-late osteoarthritis, RA-rheumatoid arthritis, SM-sphingomyelin, Cer-ceramide, HexCer-hexosylceramide (most likely glucosylceramide), Hex2Cer-dihexosylceramide (most likely lactosylceramide), PA-phospatidic acid, LPA-lysophosphatidic acid, PG-phosphatidylglycerol, LPG-lysophosphatidylglycerol, SPH-sphingosine, SPC-sphingosylphosphorylcholine, CL-cardiolipin, BMP-bis(monoglycero)phosphate. (PDF) [file pone.0091769.s004.pdf]

Table S1. Concentrations of lipid species presented in Figure 1-7.

Lipid	Control (n=9) [nmol/ml]	eOA (n=17) [nmol/ml]	lOA (n=13) [nmol/ml]	RA (n=18) [nmol/ml]
**SM 34:0**	**0.45 (0.40-0.64)**	**1.24 (0.80-1.32)**	**1.42 (1.26-3.85)**	**1.71 (1.42-2.39)**
	0.45 (0.40-0.64)	0.86 (0.78-1.35)	1.02 (0.94-1.20)	1.93 (1.58-2.56)
**SM 36:0**	**0.11 (0.06-0.13)**	**0.16 (0.08-0.34)**	**0.34 (0.23-0.54)**	**0.37 (0.25-0.52)**
	0.11 (0.06-0.13)	0.15 (0.05-0.29)	0.23 (0.14-0.35)	0.40 (0.33-0.69)
**SM 32:1**	**1.08 (0.76-1.54)**	**3.06 (1.83-4.60)**	**5.17 (3.58-11.5)**	**5.42 (4.40-5.93)**
	1.08 (0.76-1.54)	2.68 (1.18-4.68)	3.36 (2.96-3.59)	5.71 (4.91-6.52)
**SM 33:1**	**0.65 (0.48-0.89)**	**1.48 (1.18-2.49)**	**2.16 (1.80-5.84)**	**2.76 (2.65-3.16)**
	0.65 (0.48-0.89)	1.25 (0.66-2.28)	1.61 (1.38-1.79)	3.30 (2.67-3.75)
**SM 34:1**	**17.0 (11.7-17.7)**	**33.6 (23.4-52.8)**	**52.6 (36.5-112.7)**	**46.4 (41.8-58.6)**
	17.0 (11.7-17.7)	28.9 (20.4-37.9)	**30.3 (27.6-33.8)**	55.2 (47.0-65.8)
**SM 35:1**	**0.15 (0.12-0.17)**	**0.33 (0.21-0.57)**	**0.60 (0.43-1.33)**	**0.43 (0.37-0.55)**
	0.15 (0.12-0.17)	0.34 (0.18-0.41)	0.36 (0.33-0.39)	0.50 (0.44-0.61)
**SM 36:1**	**2.13 (0.88-2.40)**	**3.85 (2.95-7.60)**	**7.86 (5.08-12.9)**	**8.24 (5.69-12.2)**
	2.13 (0.88-2.40)	3.55 (2.38-5.25)	4.44 (3.62-5.22)	10.0 (7.53-12.6)
**SM 38:1**	**0.29 (0.15-0.54)**	**0.90 (0.50-1.59)**	**1.92 (1.46-3.31)**	**1.88 (0.99-3.96)**
	0.29 (0.15-0.54)	1.04 (0.40-1.49)	1.40 (0.73-1.75)	2.48 (0.98-3.48)
**SM 40:1**	**0.77 (0.30-1.27)**	**2.11 (1.64-4.23)**	**4.29 (3.14-8.88)**	**4.71 (3.07-8.09)**
	0.77 (0.30-1.27)	1.98 (1.22-3.50)	2.77 (2.06-3.11)	6.27 (3.78-7.74)
**SM 42:1**	**0.89 (0.65-1.33)**	**2.71 (1.66-4.15)**	**3.99 (3.05-9.17)**	**4.64 (3.62-6.70)**
	0.89 (0.65-1.33)	2.17 (1.44-3.48)	2.65 (2.10-3.03)	5.61 (4.02-7.47)
**SM 34:2**	**2.18 (1.42-2.35)**	**4.34 (3.11-7.47)**	**7.20 (5.28-13.8)**	**6.02 (5.11-6.72)**
	2.18 (1.42-2.35)	3.48 (2.71-5.40)	4.15 (3.68-4.68)	6.64 (5.99-7.91)
**SM 35:2**	**0.35 (0.25-0.47)**	**0.78 (0.61-1.09)**	**1.20 (0.86-3.12)**	**1.37 (1.17-1.58)**
	0.35 (0.25-0.47)	0.65 (0.37-1.03)	0.76 (0.63-0.91)	1.56 (1.37-1.76)
**SM 36:2**	**0.99 (0.50-1.17)**	**1.70 (1.47-3.15)**	**3.42 (2.14-4.99)**	**3.43 (2.11-4.20)**
	0.99 (0.50-1.17)	1.54 (1.12-2.38)	1.95 (1.59-2.29)	3.81 (3.16-4.34)
**SM 38:2**	**0.54 (0.27-0.56)**	**0.91 (0.72-1.57)**	**1.66 (1.20-2.47)**	**1.68 (1.05-1.99)**
	0.54 (0.27-0.56)	0.73 (0.57-1.23)	0.98 (0.79-1.23)	1.88 (1.46-2.10)
**SM 40:2**	**1.51 (0.80-1.85)**	**3.25 (2.15-5.92)**	**6.42 (4.11-10.6)**	**6.22 (4.33-7.78)**
	1.51 (0.80-1.85)	2.64 (1.88-4.12)	3.58 (2.77-4.17)	7.06 (5.64-8.24)
**SM 42:2**	**4.91 (3.46-5.87)**	**10.4 (6.99-15.4)**	**16.5 (11.7-33.0)**	**16.7 (13.3-21.0)**
	4.91 (3.46-5.87)	8.81 (6.00-13.9)	9.52 (8.65-11.6)	18.8 (14.4-25.4)
**SM 40:3**	**0.18 (0.16-0.24)**	**0.33 (0.16-0.54)**	**0.54 (0.38-1.07)**	**0.66 (0.50-0.86)**
	0.18 (0.16-0.24)	0.22 (0.13-0.57)	0.38 (0.20-0.51)	0.74 (0.56-0.90)
**SM 42:3**	**3.21 (2.12-3.32)**	**5.79 (4.19-9.21)**	**10.1 (6.17-17.9)**	**8.63 (6.47-10.2)**
	3.21 (2.12-3.32)	4.77 (3.49-7.36)	5.43 (4.66-5.93)	10.4 (7.28-11.2)
**SM 42:4**	**0.28 (0.19-0.46)**	**0.51 (0.34-0.86)**	**0.54 (0.48-2.00)**	**0.64 (0.42-0.70)**
	0.28 (0.19-0.46)	0.45 (0.28-0.64)	0.43 (0.42-0.51)	0.72 (0.57-0.79)
**Cer d18:1/16:0**	**0.21 (0.17-0.33)**	**0.32 (0.22-0.47)**	**0.45 (0.34-1.08)**	**0.54 (0.43-0.70)**
	0.21 (0.17-0.33)	0.26 (0.17-0.38)	0.28 (0.24-0.35)	0.61 (0.53-0.74)
**Cer d18:1/18:0**	**0.06 (0.05-0.10)**	**0.08 (0.06-0.10)**	**0.09 (0.05-0.31)**	**0.08 (0.06-0.10)**
	0.06 (0.05-0.10)	0.06 (0.05-0.08)	0.05 (0.04-0.06)	0.10 (0.07-0.11)

**Cer d18:1/22:0**	**0.12 (0.08-0.15)**	**0.17 (0.11-0.30)**	**0.28 (0.23-0.53)**	**0.35 (0.21-0.51)**
	0.12 (0.08-0.15)	0.13 (0.09-0.20)	0.18 (0.16-0.22)	0.45 (0.33-0.49)
**Cer d18:1/23:0**	**0.17 (0.16-0.24)**	**0.32 (0.17-0.44)**	**0.39 (0.32-0.92)**	**0.56 (0.36-0.72)**
	0.17 (0.16-0.24)	0.24 (0.15-0.33)	0.25 (0.22-0.35)	0.69 (0.48-0.85)
**Cer d18:1/24:0**	**0.31 (0.28-0.39)**	**0.72 (0.47-1.26)**	**1.23 (1.04-2.24)**	**1.54 (0.99-2.08)**
	0.31 (0.28-0.39)	0.60 (0.41-1.08)	0.74 (0.58-1.02)	1.97 (1.33-2.15)
**Cer d18:1/24:1**	**0.38 (0.34-0.46)**	**0.61 (0.43-0.95)**	**0.93 (0.64-1.60)**	**1.10 (0.82-1.32)**
	0.38 (0.34-0.46)	0.45 (0.33-0.69)	0.62 (0.46-0.71)	1.22 (1.03-1.38)
**HexCer d18:1/16:0**	**57.4 (43.1-74.1)**	**132.4 (86.6-239.1)**	**202.6 (168.1-351.3)**	**364.3 (262.5-418.6)**
	57.4 (43.1-74.1)	126.9 (71.4-210.4)	132.6 (101.0-183.6)	411.6 (301.0-526.3)
**HexCer d18:1/22:0**	**56.6 (33.8-94.0)**	**198.1 (114.1-321.2)**	**220.2 (198.1-479.9)**	**326.6 (226.5-377.1)**
	56.6 (33.8-94.0)	168.7 (87.4-263.6)	143.8 (121.3-223.1)	366.1 (282.8-495.2)
**HexCer d18:1/23:0**	**25.8 (17.1-45.4)**	**95.0 (55.5-156.7)**	**126.9 (105.7-251.3)**	**188.4 (128.7-229.2)**
	25.8 (17.1-45.4)	86.3 (47.2-142.1)	77.7 (57.2-124.9)	219.1 (160.0-276.7)
**HexCer d18:1/24:0**	**83.8 (54.7-151.5)**	**305.8 (172.6-465.8)**	**345.6 (300.8-683.2)**	**510.1 (398.0-687.4)**
	83.8 (54.7-151.5)	274.0 (150.2-381.0)	238.7 (178.7-328.6)	622.9 (424.6-816.3)
**HexCer d18:1/24:1**	**114.3 (82.1-141.1)**	**282.6 (145.5-472.7)**	**397.2 (282.6-609.7)**	**546.9 (358.5-702.1)**
	114.3 (82.1-141.1)	256.6 (139.5-412.6)	250.5 (205.9-314.7)	660.4 (445.2-877.0)
**Hex_2Cer d18:1/16:0**	**186.8 (85.6-262.2)**	**432.4 (297.4-572.0)**	**511.1 (364.3-1199.8)**	**1189.3 (788.0-1328.1)**
	186.8 (85.6-262.2)	433.2 (250.5-673.8)	377.2 (266.4-491.5)	1465.4 (997.0-1720.2)
**Hex_2Cer d18:1/22:0**	**16.5 (7.60-18.8)**	**28.2 (23.9-50.5)**	**31.4 (24.5-76.1)**	**77.9 (43.3-92.0)**
	16.5 (7.60-18.8)	27.1 (20.4-44.5)	27.1 (17.4-35.1)	86.1 (64.2-108.4)
**Hex_2Cer d18:1/23:0**	**4.55 (0.79-5.10)**	**9.89 (7.15-17.1)**	**10.7 (7.08-19.2)**	**20.4 (12.2-23.8)**
	4.55 (0.79-5.10)	10.3 (6.24-14.7)	7.54 (5.06-12.0)	26.4 (14.5-28.5)
**Hex_2Cer d18:1/24:0**	**16.1 (12.0-19.5)**	**38.4 (25.5-49.9)**	**39.6 (30.1-94.3)**	**100.1 (56.1-117.9)**
	16.1 (12.0-19.5)	33.8 (24.5-67.0)	28.6 (21.5-41.2)	122.2 (93.2-142.2)
**Hex_2Cer d18:1/24:1**	**64.3 (35.8-90.3)**	**126.0 (96.8-167.4)**	**151.9 (99.8-314.6)**	**329.6 (281.9-437.1)**
	64.3 (35.8-90.3)	119.5 (79.8-198.3)	108.6 (65.6-142.3)	450.9 (351.2-597.5)
**PA 34:1**	**0.37 (0.18-0.50)**	**0.54 (0.36-1.29)**	**1.08 (0.82-2.11)**	**1.19 (0.78-1.54)**
	0.37 (0.18-0.50)	0.61 (0.34-0.88)	0.61 (0.49-1.04)	1.33 (1.08-1.91)
**PA 36:1**	**0.15 (0.06-0.22)**	**0.23 (0.17-0.49)**	**0.46 (0.27-0.78)**	**0.83 (0.56-1.45)**
	0.15 (0.06-0.22)	0.22 (0.16-0.33)	0.25 (0.18-0.37)	1.16 (0.71-1.58)
**PA 36:2**	**0.27 (0.11-0.35)**	**0.47 (0.27-1.42)**	**1.14 (0.69-1.80)**	**1.24 (0.78-1.63)**
	0.27 (0.11-0.35)	0.46 (0.25-0.96)	0.59 (0.48-0.79)	1.47 (1.22-2.16)
**PA 38:4**	**0.03 (0.00-0.10)**	**0.16 (0.00-0.42)**	**0.22 (0.18-0.47)**	**0.35 (0.22-0.47)**
	0.03 (0.00-0.10)	0.14 (0.00-0.29)	0.17 (0.12-0.23)	0.43 (0.25-0.56)
**LPA 16:0**	**0.27 (0.07-0.54)**	**0.71 (0.50-1.70)**	**1.37 (0.93-2.49)**	**1.26 (0.87-2.78)**
	0.27 (0.07-0.54)	0.64 (0.46-1.27)	0.89 (0.61-1.45)	1.64 (1.22-3.43)
**LPA 18:0**	**0.10 (0.00-0.11)**	**0.18 (0.08-0.37)**	**0.20 (0.13-0.33)**	**0.25 (0.16-0.44)**
	0.10 (0.00-0.11)	0.12 (0.10-0.28)	0.16 (0.08-0.24)	0.31 (0.22-0.56)

**LPA 18:1**	**0.31 (0.08-0.41)**	**0.45 (0.32-1.18)**	**0.87 (0.52-1.17)**	**0.75 (0.52-1.17)**
	0.31 (0.08-0.41)	0.40 (0.30-0.88)	0.46 (0.31-0.82)	0.82 (0.75-1.33)
**LPA 18:2**	**0.87 (0.15-2.22)**	**2.36 (1.80-7.09)**	**5.21 (2.44-6.34)**	**3.34 (1.61-4.94)**
	0.87 (0.15-2.22)	2.75 (1.82-6.76)	2.57 (1.31-3.63)	3.97 (2.94-5.33)
**LPA 20:4**	**2.84 (1.19-5.02)**	**5.11 (3.66-10.7)**	**9.46 (5.24-11.8)**	**7.22 (4.40-10.7)**
	2.84 (1.19-5.02)	4.72 (4.01-7.24)	5.37 (2.86-8.26)	8.18 (6.67-12.3)
**PG 34:1**	**0.22 (0.09-0.44)**	**0.42 (0.33-0.54)**	**0.58 (0.31-1.43)**	**0.80 (0.58-0.92)**
	0.22 (0.09-0.44)	0.43 (0.33-0.49)	0.40 (0.30-0.44)	0.92 (0.68-1.16)
**PG 36:1**	**0.18 (0.10-0.24)**	**0.48 (0.37-0.53)**	**0.48 (0.36-1.27)**	**0.72 (0.53-0.98)**
	0.18 (0.10-0.24)	0.39 (0.34-0.54)	0.39 (0.27-0.46)	0.88 (0.69-1.20)
**PG 36:2**	**0.34 (0.29-0.49)**	**0.66 (0.43-0.76)**	**0.68 (0.43-2.66)**	**0.80 (0.65-0.90)**
	0.34 (0.29-0.49)	0.61 (0.51-0.66)	0.54 (0.48-0.61)	1.01 (0.78-1.17)
**PG 36:3**	**0.00 (0.00-0.08)**	**0.15 (0.11-0.19)**	**0.12 (0.05-0.15)**	**0.20 (0.16-0.23)**
	0.00 (0.00-0.08)	0.15 (0.12-0.16)	0.10 (0.07-0.13)	0.23 (0.19-0.29)
**PG 38:4**	**0.00 (0.00-0.00)**	**0.08 (0.00-0.14)**	**0.05 (0.00-0.11)**	**0.14 (0.10-0.15)**
	0.00 (0.00-0.00)	0.11 (0.00-0.12)	0.06 (0.00-0.09)	0.17 (0.13-0.27)
**LPG 16:0**	**0.28 (0.05-0.44)**	**0.29 (0.22-0.51)**	**0.34 (0.10-0.66)**	**0.45 (0.36-0.52)**
	0.28 (0.05-0.44)	0.30 (0.22-0.37)	0.28 (0.12-0.46)	0.53 (0.41-0.67)
**LPG 18:1**	**1.69 (0.96-2.44)**	**1.43 (1.05-1.73)**	**1.82 (1.00-2.50)**	**1.21 (1.06-1.56)**
	1.69 (0.96-2.44)	1.20 (1.07-1.67)	1.11 (1.00-1.34)	1.50 (1.27-1.75)
**SPH d18:0**	**0.00 (0.00-0.00)**	**0.00 (0.00-0.00)**	**0.00 (0.00-0.00)**	**11.8 (0.00-47.6)**
	0.00 (0.00-0.00)	0.00 (0.00-0.00)	0.00 (0.00-0.00)	0.00 (0.00-68.2)
**SPH d18:1**	**0.00 (0.00-0.00)**	**0.00 (0.00-0.00)**	**0.00 (0.00-0.00)**	**27.3 (18.3-40.2)**
	0.00 (0.00-0.00)	0.00 (0.00-0.00)	0.00 (0.00-0.00)	39.4 (27.0-55.5)
**SPC d18:0**	**0.00 (0.00-0.00)**	**0.00 (0.00-0.00)**	**0.00 (0.00-0.00)**	**0.00 (0.00-1.79)**
	0.00 (0.00-0.00)	0.00 (0.00-0.00)	0.00 (0.00-0.00)	0.00 (0.00-2.32)
**SPC d18:1**	**0.00 (0.00-1.66)**	**7.49 (0.00-17.5)**	**9.35 (4.30-12.3)**	**16.1 (10.8-31.2)**
	0.00 (0.00-1.66)	8.44 (0.00-12.8)	8.82 (5.02-9.81)	21.1 (11.6-50.1)
**CL 36:4/36:4**	**0.00 (0.00-0.00)**	**0.00 (0.00-0.00)**	**0.00 (0.00-0.00)**	**0.02 (0.01-0.03)**
	0.00 (0.00-0.00)	0.00 (0.00-0.00)	0.00 (0.00-0.00)	0.02 (0.01-0.03)
**BMP 18:1/18:1**	**0.37 (0.23-0.62)**	**0.31 (0.21-0.50)**	**0.49 (0.31-1.00)**	**0.32 (0.26-0.41)**
	0.37 (0.23-0.62)	0.33 (0.21-0.46)	0.30 (0.25-0.42)	0.42 (0.33-0.53)
**BMP 18:1/18:2**	**0.21 (0.12-0.27)**	**0.22 (0.15-0.34)**	**0.34 (0.21-0.62)**	**0.25 (0.19-0.31)**
	0.21 (0.12-0.27)	0.22 (0.12-0.33)	0.21 (0.18-0.25)	0.30 (0.24-0.39)

Lipids were determined by electrospray ionization tandem mass spectrometry or liquid chromatography coupled with tandem mass spectrometry as outlined in *Material and*

Methods. Data (nmol/ml or pmol/ml) obtained were either uncorrected (normal font) or corrected with the dilution factor per Kraus *et al.* (34, bold font). Values are median and interquartile range. The concentrations of urea were determined within serum and SF to calculate a dilution factor for SF; this procedure was formerly developed to adjust for the dilution introduced by lavage during some biomarker studies. eOA-early osteoarthritis, lOA-late osteoarthritis, RA-rheumatoid arthritis, SM-sphingomyelin, Cer-ceramide, HexCer-hexosylceramide (most likely glucosylceramide), Hex_2Cer-dihexosylceramide (most likely lactosylceramide), PA-phospatidic acid, LPA-lysophosphatidic acid, PG-phosphatidylglycerol, LPG-lysophosphatidylglycerol, SPH-sphingosine, SPC-sphingosylphosphorylcholine, CL-cardiolipin, BMP-bis(monoglycero)phosphate.
